# The effect of medial open wedge high tibial osteotomy on the patellofemoral joint: comparative analysis according to the preexisting cartilage status

**DOI:** 10.1186/s12891-019-2989-y

**Published:** 2019-12-14

**Authors:** Hyun-Soo Moon, Chong-Hyuk Choi, Min Jung, Sang-Hoon Park, Dae-Young Lee, Jong-Kwan Shin, Sung-Hwan Kim

**Affiliations:** 1 Arthroscopy and Joint Research Institute, Yonsei University College of Medicine, Seoul, Republic of Korea; 2 Department of Orthopedic Surgery, Gangnam Severance Hospital, Yonsei University College of Medicine, Seoul, Republic of Korea; 3 Department of Orthopedic Surgery, Severance Hospital, Yonsei University College of Medicine, Seoul, Republic of Korea; 4 Department of Orthopedic Surgery, National Health Insurance Service Ilsan Hospital, Gyeonggi-do, Republic of Korea; 5 Department of Orthopedic Surgery, Saegil Hospital, Seoul, Republic of Korea

**Keywords:** Knee, High tibial osteotomy, Patellofemoral joint, Cartilage lesion

## Abstract

**Background:**

Although it has been known that medial open wedge high tibial osteotomy (MOWHTO) would adversely affect the patellofemoral joint, no previous study examined the surgical outcome of MOWHTO according to the preexisting cartilage status of the patellofemoral joint. The aim of this study was to investigate the effect of MOWHTO on the patellofemoral joint with regard to objective and subjective aspects according to the preexisting cartilage status.

**Methods:**

Ninety-two patients who underwent MOWHTO and a following second-look arthroscopic assessment were included in this study. The patients were divided into two groups according to the preexisting cartilage status of the patellofemoral joint: group 1 (International Cartilage Repair Society [ICRS] grade 2 or 3) and group 2 (ICRS grade 0 or 1). Comparative analysis was performed regarding clinical scores, radiographic parameters, and arthroscopic measurements between the two groups.

**Results:**

Clinical outcomes showed overall improvement from baseline to the time of second-look operation, with no significant difference between the two groups at each time point. There were no significant differences in radiographic parameters between the two groups. Radiographic grade of patellofemoral osteoarthritis in both groups showed a tendency to progress, without statistical significance. In arthroscopic assessment, the size of the cartilage lesion on the patellofemoral joint increased with time in both groups (*P* = 0.003), but the degree of change over time between the two groups was not statistically significant. Consistently, there was no significant difference in the frequency of progression of cartilage lesion grade in the patellofemoral joint between the two groups.

**Conclusions:**

MOWHTO would contribute to osteoarthritis progression of the patellofemoral joint regardless of the preexisting cartilage status, without an association with clinical outcomes in short-term follow-up.

## Background

Medial open wedge high tibial osteotomy (MOWHTO) is an effective surgical procedure for the treatment of medial compartment osteoarthritis of the knee as well as for the correction of lower extremity malalignment [1–3]. With favorable clinical outcomes and improved surgical techniques, MOWHTO has become increasingly popular [4–6].

Although numerous advantages of MOWHTO have been addressed, it has been reported that MOWHTO would adversely affect the patellofemoral joint. Several studies state that MOWHTO leads to patella baja, subsequently causing increased patellofemoral contact pressure [7–9]. Varus-valgus alignment was also reported to affect the progression of patellofemoral osteoarthritis in a compartment-specific manner [10]. Recently, several studies investigating the effect of MOWHTO on the patellofemoral joint using an arthroscopic assessment also reported overall deterioration of the articular cartilage of the patellofemoral joint over time as a result of MOWHTO [11–14].

However, it is difficult to conclude that MOWHTO definitely contributes to the deterioration of the articular cartilage of the patellofemoral joint. There are various factors that could affect the progression of the patellofemoral osteoarthritis [15]. In particular, the possibility of the normal progression of preexisting cartilage lesions could not be ruled out. Focal cartilage lesions of the joints are known to cause stress concentration in the rim of the defect, acting as a leading factor of arthritis [16]. Due to the nature of the study design of the abovementioned studies [11–14], which assessed articular cartilage status arthroscopically, could not provide details according to the presence or absence of preexisting articular cartilage lesions. To determine the contribution of MOWHTO to the progression of patellofemoral joint osteoarthritis, the effect of preexisting cartilage lesions should be investigated. To the best of the authors’ knowledge, no previous study examined the outcome of MOWHTO according to the preexisting cartilage status of the patellofemoral joint.

The purpose of this study was to investigate the effect of MOWHTO on the patellofemoral joint with regard to the objective and subjective aspects according to the preexisting cartilage status. The hypothesis was as follows: (1) MOWHTO would contribute to progression of patellofemoral joint osteoarthritis regardless of the presence of preexisting cartilage lesions in the objective perspective and (2) clinical outcome of MOWHTO in patients with preexisting cartilage lesion of patellofemoral joint would not be inferior compared to those with normal patellofemoral joint cartilage in the subjective perspective.

## Methods

**Fig. 1:**
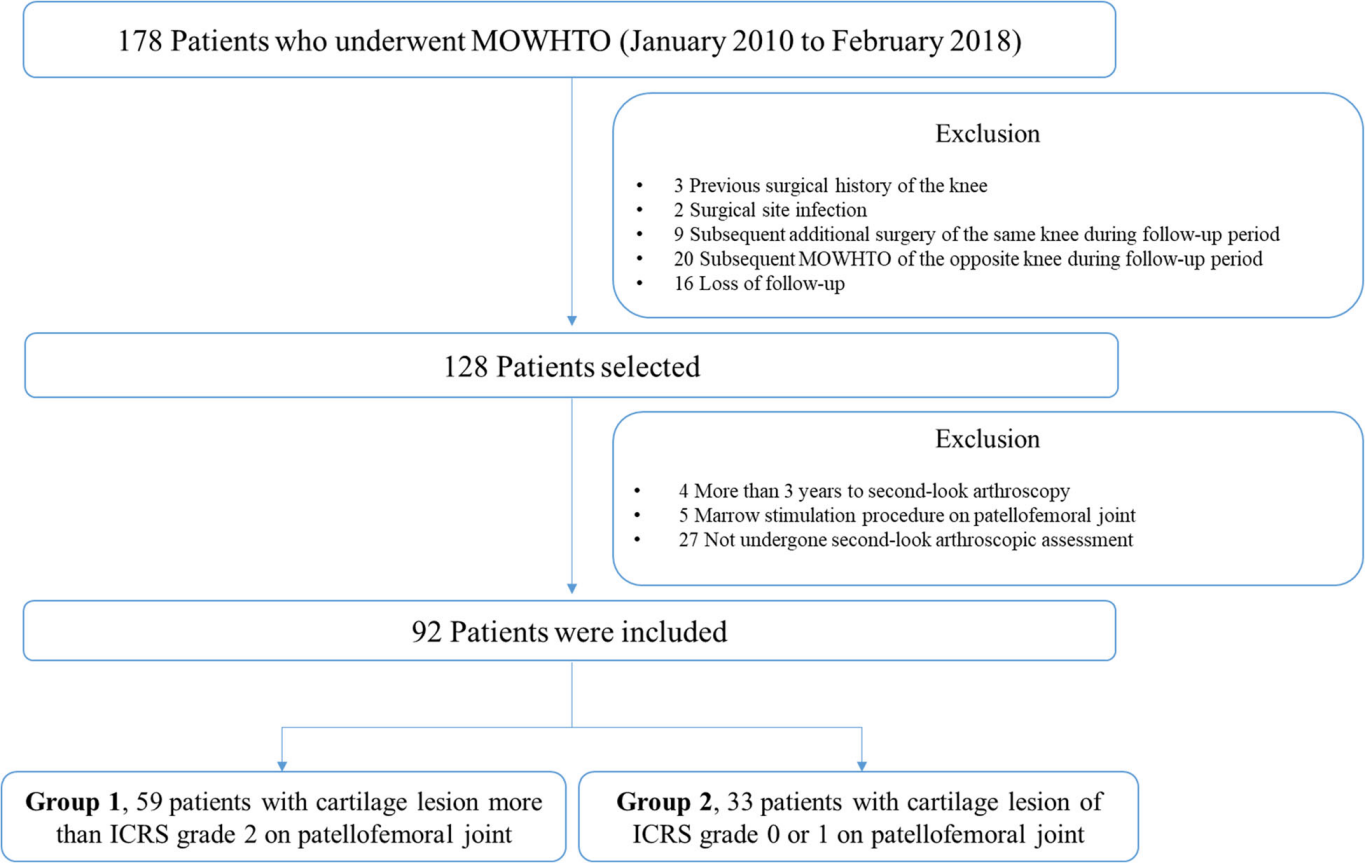
Flowchart of patient inclusion in the study

This study was approved by the institutional review board prior to the study, which waived the requirement for informed consent from patients owing to the retrospective nature of the study. Data of 178 consecutive patients, who underwent biplane MOWHTO by a single orthopedic surgeon in a single institution between January 2010 and February 2018 were reviewed retrospectively. Of those, patients who underwent second-look arthroscopic assessment were eligible to be included in this study. The exclusion criteria were as follows: (1) history of previous surgical treatment of the knee, (2) surgical site infection, (3) additional surgical procedure of the same knee during the follow-up period, and (4) the same subsequent surgical procedure of the opposite knee during the follow-up period. In addition, the patients with a time more than 3 years from MOWHTO to second-look operation, and the patients who underwent marrow stimulation procedure on the International Cartilage Repair Society (ICRS) grade 4 cartilage lesion in patellofemoral joint at initial operation were excluded to increase comparability. As a result, a total of 92 cases who met abovementioned conditions were included in this study and divided into 2 groups according to the preexisting cartilage status of the patellofemoral joint. The distribution of patients was as follows: (1) group 1, 59 patients with patellofemoral joint cartilage lesion greater than ICRS grade 2 and (2) group 2, 33 patients with cartilage lesion of ICRS grade 0 or 1 (Fig. 1). For the cartilage lesions in both the patella and trochlea, higher grade lesions were used as a reference. Baseline characteristics were similar between the two groups (Table 1). The mean time from MOWHTO to the second-look operation was 21.7 ± 6.2 and 21.2 ± 5.3 months for group 1 and 2, respectively. In addition, subgroup analysis of group 1, revealed that there were no statistically significant within-group differences (Additional file 1).

**Table 1:** Comparison of baseline characteristics

Variable	Group 1 (*n* = 59)	Group 2 (*n* = 33)	*P* value
Age ^a^ (year)	54.3 ± 5.2	55.5 ± 4.4	0.261
Gender ^b^			0.745
Male	16 (27.1%)	10 (30.3%)	
Female	43 (72.9%)	23 (69.7%)	
BMI ^a^ (kg/m2)	26.2 ± 5.9	25.6 ± 5.2	0.613
Load-bearing axis deviation ^a^ (%)	19.0 ± 11.7	19.3 ± 10.5	0.905
Hip-Knee-Ankle angle ^a^ (varus,°)	7.9 ± 2.9	7.7 ± 2.7	0.762
Medial proximal tibial angle ^a^ (°)	84.0 ± 2.2	84.1 ± 1.9	0.843
Joint line convergence angle ^a^ (°)	3.2 ± 1.7	2.9 ± 1.7	0.437
Posterior tibial slope (°)	5.7 ± 3.2	6.2 ± 4.0	0.590
Blackburne-Peel ratio ^a^	0.9 ± 0.2	0.9 ± 0.2	0.186
Caton-Deschamps ratio ^a^	1.0 ± 0.2	1.1 ± 0.2	0.132
Lateral patellofemoral angle ^a^ (°)	13.9 ± 4.5	12.3 ± 4.3	0.098
Trochlear dysplasia grade according to the Dejour classification^b^			0.547
No	56 (94.9%)	32 (97.0%)	
Type A	3 (5.1%)	1 (25.0%)	
Kellgren–Lawrence grade^b^			0.637
Grade 1	0 (0.0%)	1 (3.0%)	
Grade 2	12 (20.3%)	7 (21.2%)	
Grade 3	37 (62.7%)	21 (63.6%)	
Grade 4	10 (16.9%)	4 (12.1%)	
Patellofemoral osteoarthritis stage according to Iwano’s classification^b^			0.096
Stage 0	10 (16.9%)	12 (36.4%)	
Stage 1	27 (45.8%)	13 (39.4%)	
Stage 2	22 (37.3%)	8 (24.2%)	
Correction angle ^a^ (°)	11.0 ± 2.1	11.3 ± 2.6	0.585
Time to hardware removal (months) ^a^	21.7 ± 6.2	21.2 ± 5.3	0.664
Preoperative VAS score ^a^	68.4 ± 17.7	63.4 ± 19.8	0.223
Preoperative IKDC subjective score ^a^	33.8 ± 11.8	36.7 ± 14.8	0.308
Preoperative Kujala score ^a^	38.3 ± 14.7	37.5 ± 16.9	0.812

*BMI* body mass index, *VAS* Visual analogue scale, *IKDC* International Knee Documentation Committee

^a^ The values are given as the mean and standard deviation

^b^ The values are given as the number of patients, with the percentage in parenthesis

### Surgical indications and procedure

Surgical indications for MOWHTO were as follows: (1) patients younger than 65 years who had medial compartment osteoarthritis with varus malalignment, (2) activity-related medial-sided knee pain, (3) good range of motion (arc of motion > 100° and flexion contracture < 15°) and without joint instability. MOWHTO was not indicated for the patients who complained of anterior knee pain associated with activities, such as squatting and stair climbing or descending. Moreover, patients with more than stage 3 of patellofemoral osteoarthritis according to Iwano’s osteoarthritis classification system were excluded regardless of symptoms [17]. All patients were recommended to perform hardware removal if bony consolidation was confirmed, due to the possible postoperative pain owing to local irritation of the plate. If the plate removal was planned, second-look arthroscopic assessment was recommended to be performed at the same time.

In all patients, preoperative surgical planning for obtaining appropriate alignment of the lower limb was performed according to the Miniaci method [18], realigning the mechanical axis to be located at the Fujisawa point [19]. Prior to the osteotomy procedure, diagnostic arthroscopy was performed, and the status of articular cartilage was evaluated thoroughly. Cartilage procedure such as debridement and chondroplasty was not performed on the cartilage lesion of patellofemoral joint. After the arthroscopic assessment, biplane MOWHTO was performed. To expose the medial proximal tibia, an approximately 6–8-cm oblique skin incision was made from 1 cm below the joint line to the pes anserinus tendons between the tibial tuberosity and the inner border of the tibia. Then, the distal superficial medial collateral ligament was released and underlying periosteum was removed. Two starting guide wires for transverse osteotomy were inserted parallel from the upper border of the pes anserinus tendons toward the upper portion of the fibular head. Prior to transverse osteotomy, the separate oblique vertical osteotomy in the coronal plane was made 1 cm behind the tibial tuberosity. Transverse osteotomy was initiated subsequently with an oscillating saw along the two guide wires leaving the lateral most at 1 cm of proximal tibia as a hinge. Osteotomy site was opened gradually using several chisels and a spreader device. After the desired correction was achieved, TomoFix plate (Synthes, West Chester, PA) was applied and fixed to the medial proximal tibia over the osteotomy site.

Postoperatively, patients were instructed to begin crutch-assisted progressive weight-bearing ambulation as tolerated. After restricting the knee range motion for 2 weeks with splint immobilization, exercise for knee range motion was initiated with hinged knee brace. Six weeks after the surgery, all patients were encouraged to remove both crutch and hinged knee brace.

### Evaluation

**Fig. 2:**
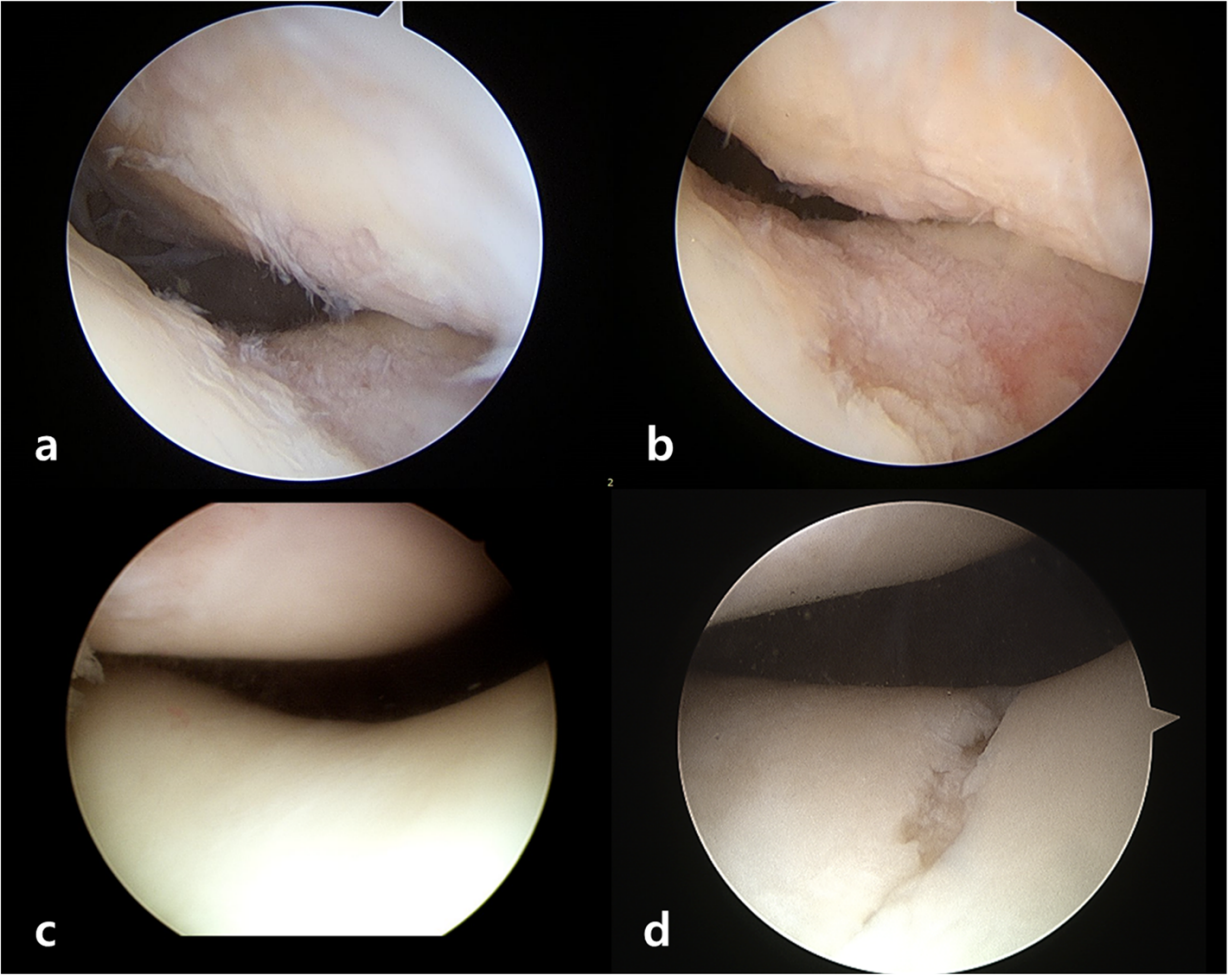
Comparison of an arthroscopic assessment at initial operation and at the time of second-look operation. **a** Patient of group 1 showed preexisting cartilage lesions on patellofemoral joint (patella; ICRS grade III cartilage lesions with about 15x15mm size, trochlea; ICRS grade III cartilage lesions with about 5x20mm size), **b** which were observed to be progressed at second-look operation (patella; ICRS grade III cartilage lesions with about 15x15mm size, trochlea; ICRS grade III cartilage lesions with about 20x25mm size). **c** Patient of group 2 without any preexisting cartilage lesions on patellofemoral joint **d** showed development of cartilage lesions at second-look operation (trochlea; ICRS grade III cartilage lesions with about 3x15mm size). *ICRS* International Cartilage Repair Society

Comparative analysis of clinical outcomes, radiographic factors, and arthroscopic measurements were performed. Clinical outcomes were assessed preoperatively and at the time of second-look operation using various patient-reported knee rating scales, including the visual analog scale (VAS) [20], International Knee Documentation Committee (IKDC) subjective score [21], and Kujala scale (anterior knee pain scale) [22]. The degree of osteoarthritis was radiographically assessed using the Kellgren-Lawrence grading system for the tibiofemoral joint and the Iwano classification system for the patellofemoral joint [17, 23]. Various radiographic parameters possibly associated with the preexisting condition of the patellofemoral joint, including load-bearing axis deviation [24], hip-knee-ankle angle [24], medial proximal tibial angle [25], joint line convergence angle [25], posterior tibial slope [26], and the presence of trochlear dysplasia [27] were evaluated. In addition, the Caton-Deschamps index and Blackburne-Peel ratio were used to assess patellar height and the lateral patellofemoral angle was used to measure patella tilt [28–30]. Two orthopedic surgeons who were not involved in the surgery measured all radiographic parameters with an interval of 6 weeks and were blinded to each other’s measurements. Arthroscopic assessment was performed at the time of the initial operation and at the time of removal of the fixed plate. The total size of the cartilage lesion for each compartment of the knee was measured using 5-mm hook portion of an arthroscopic probe, and the severity of cartilage lesion was evaluated according to ICRS grading system [31] (Fig. 2a, b). All arthroscopic measurements were recorded immediately after the surgery by the orthopedic surgeon who performed the MOWHTO, and the assessments related to arthroscopic findings used in this study were based on this record.

### Statistical analysis

All statistical analyses were conducted using IBM SPSS Statistics version 23.0 (IBM Corp., Released 2015, Armonk, NY, USA). A non-inferiority test was performed by examining whether the 95% confidence interval (CI) for the difference of Kujala score assessed at the time of second-look operation between the two groups was less than the non-inferiority margin. The Kujala scale was set as a basis of comparison regarding clinical outcomes, since it is known as a valid and reliable scale for discriminating the differences in the severity of anterior knee pain [22]. The non-inferiority margin was set as 14, which is the reported value of minimal detectable change of the Kujala score [32]. In addition, the sample size was calculated on the basis of the abovementioned non-inferiority test. The reference value of the standard deviation (15.1) was adopted from a previous study [33]. By setting the significance level (alpha) at 5% and power (1-beta) at 90%, a minimum of 20 patients in each group turned out to be adequate to detect the difference between the two groups.

Bivariate analysis was performed to compare the baseline characteristics, clinical outcomes, and radiographic outcomes between the groups. Student’s t-test and Mann-Whitney U test were used for continuous variables, while Pearson’s chi-squared and Fisher’s exact tests were performed to evaluate categorical variables. To investigate the difference of change over time in the size of cartilage lesions between the two groups, repeated measures analysis of variance test was used. Wilcoxon-signed rank test was performed to compare the osteoarthritis grade of the patellofemoral joint and the grade of cartilage lesions at two separate time points. The kappa coefficient was used to evaluate the reliability for the evaluation of radiographic osteoarthritis grade [34], which revealed that inter-rater agreement regarding the radiographic osteoarthritis grade of the tibiofemoral joint and patellofemoral joint were 0.813 and 0.735 at preoperative time, respectively, and 0.781 and 0.761 at the time of second-look operation, respectively. A *P* value < 0.05 was considered statistically significant*.*

## Results

**Fig. 3:**
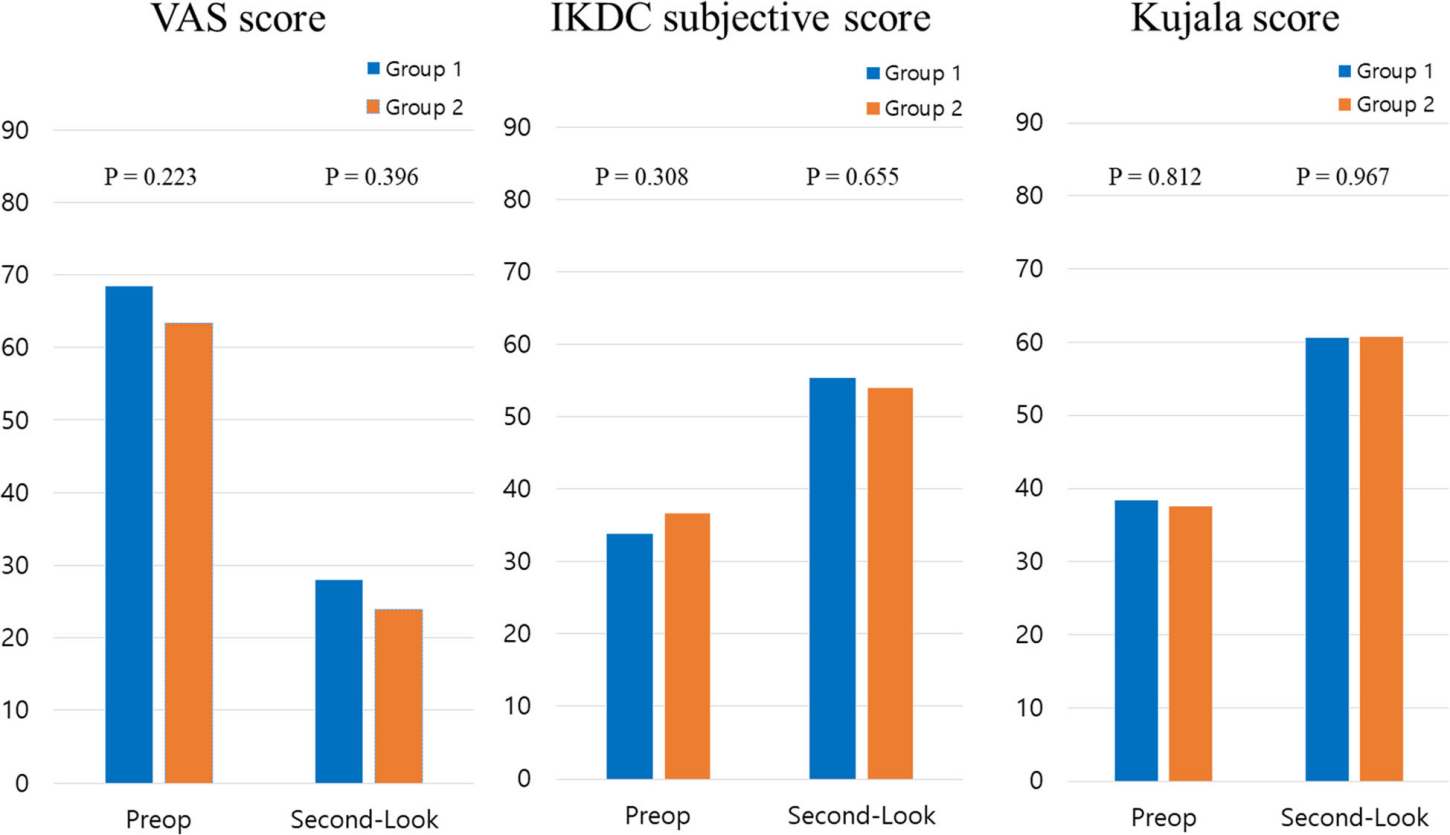
Comparison of clinical scores at preoperatively and at the time of second-look operation. *VAS* Visual analogue scale, *IKDC* International Knee Documentation Committee, *Preop* preoperative

Clinical results assessed using VAS, IKDC subjective, and Kujala scores showed overall improvement from baseline to the time of second-look operation, with no significant difference between the two groups at each time point (Fig. 3). The 95% CI of the difference of Kujala score assessed at the time of second-look operation ranged from − 7.88 to 7.56, which did not exceed the non-inferiority margin of 14 points. Accordingly, clinical outcomes regarding anterior knee pain of group 1 was not statistically inferior to that of group 2.

There were no significant differences between the two groups regarding radiographic factors assessed at the preoperative time as well as at the time of second-look operation (Tables 1, 2). Comparing the osteoarthritis grade of the patellofemoral joint at second-look operation with the initial operation, both groups showed a tendency to progress, but without statistical significance (Table 3).

**Table 2:** Comparison of radiographic parameters at the time of second-look operation

Variable	Group 1 (*n* = 59)	Group 2 (*n* = 33)	*P* value
Load-bearing axis deviation ^a^ (%)	64.3 ± 9.2	64.2 ± 6.4	0.983
Hip-Knee-Ankle angle ^a^ (valgus, °)	4.3 ± 2.6	4.0 ± 1.7	0.542
Medial proximal tibial angle ^a^ (°)	93.7 ± 3.0	93.5 ± 2.4	0.807
Joint line convergence angle ^a^ (°)	1.8 ± 2.2	1.7 ± 2.0	0.846
Posterior tibial slope (°)	6.8 ± 4.6	7.7 ± 4.0	0.346
Blackburne-Peel ratio ^a^	0.7 ± 0.1	0.8 ± 0.2	0.146
Caton-Deschamps ratio ^a^	0.8 ± 0.2	0.9 ± 0.2	0.107
Lateral patellofemoral angle ^a^ (°)	13.5 ± 4.2	12.3 ± 5.6	0.258
Kellgren–Lawrence grade^b^			0.441
Grade 1	0 (0.0%)	1 (3.0%)	
Grade 2	8 (13.6%)	7 (21.2%)	
Grade 3	43 (72.9%)	21 (63.6%)	
Grade 4	8 (13.6%)	4 (12.1%)	
Patellofemoral osteoarthritis stage according to Iwano’s classification^b^			0.124
Stage 0	8 (13.6%)	10 (30.3%)	
Stage 1	25 (42.4%)	13 (39.4%)	
Stage 2	26 (44.1%)	10 (30.3%)	

^a^ The values are given as the mean and standard deviation

^b^ The values are given as the number of patients, with the percentage in parenthesis

**Table 3:** Assessment of radiographic osteoarthritis grade of patellofemoral joint according to Iwano’s classification system at second-look operation compared with the initial operation

	Group 1 (*n* = 59)	Group 2 (*n* = 33)	*P* value ^b^
Preoperative ^a^			0.096
Stage 0	10 (16.9%)	12 (36.4%)	
Stage 1	27 (45.8%)	13 (39.4%)	
Stage 2	22 (37.3%)	8 (24.2%)	
Postoperative (Second-Look) ^a^			0.124
Stage 0	8 (13.6%)	10 (30.3%)	
Stage 1	25 (42.4%)	13 (39.4%)	
Stage 2	26 (44.1%)	10 (30.3%)	
*P* value ^c^	0.109	0.157	

^a^ The values are given as the number of patients, with the percentage in parenthesis

^b^ Statistical significance was evaluated using Pearson’s Chi-squared or Fisher’s exact test

^c^ Statistical significance was evaluated compared with the preoperative measurement values using Wilcoxon signed rank test

**Fig. 4:**
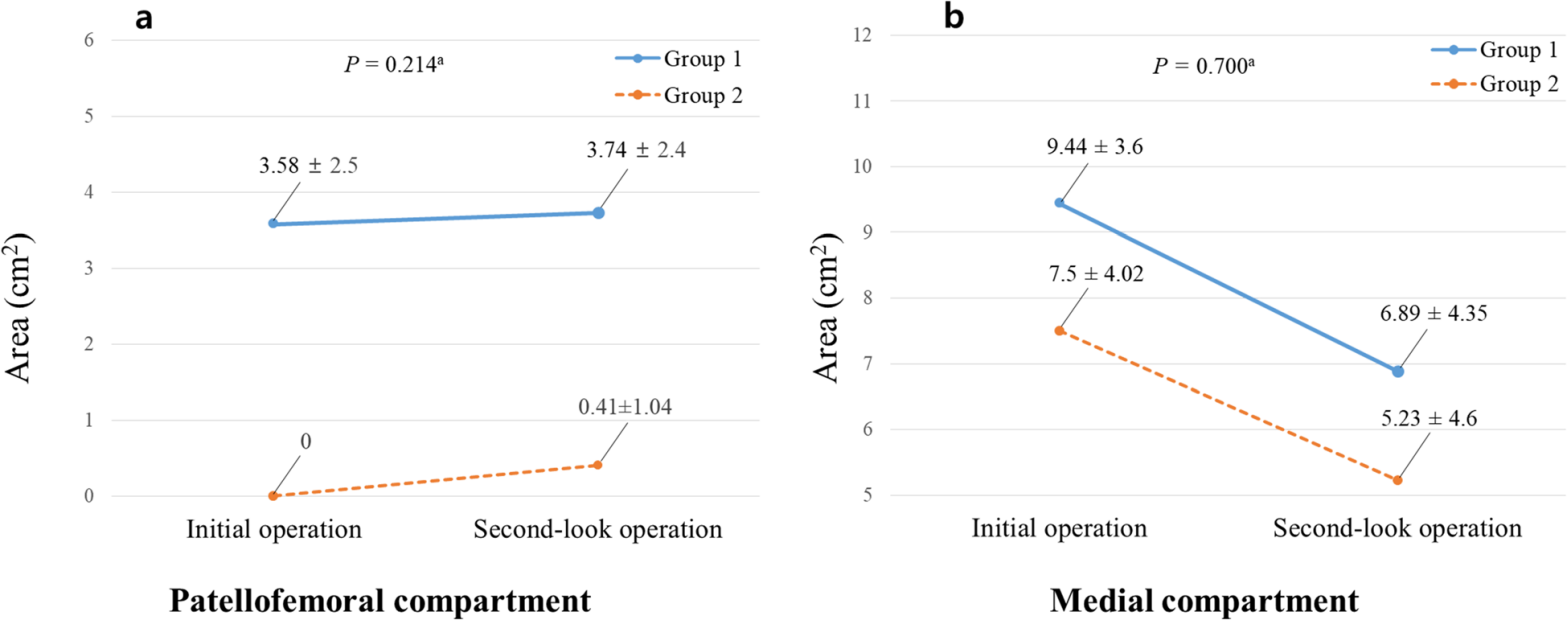
Change of the cartilage lesion size over time in each compartment of the knee. **a** the patellofemoral joint, **b** the medial compartment. ^a^ Group-by-time interaction determined using repeated measures analysis of variance

Comparison of the size and grade of cartilage lesions between the initial and second-look operations was analyzed. The size of cartilage lesion of the patellofemoral joint increased with time in both groups (*P* = 0.003). However, group-by-time interaction between the two groups was not statistically significant, indicating that the degree of change did not differ between the two groups (Fig. 4a). Consistently, there was no statistically significant difference in the frequency of progression of the cartilage lesion grade between the two groups (Table 4). Comparison of the cartilage lesion grade between two time points showed a tendency to deteriorate in both groups, but that of group 1 was not statistically significant whereas that of group 2 was statistically significant (*P* = 0.071 for group 1 and *P* = 0.007 for group 2) (Table 5). In the medial compartment of the knee, the size of the cartilage lesions in both groups decreased over time (*P* = 0.000), whereas group-by-time interaction was not statistically significant (Fig. 4b). The macroscopic regeneration staging system of Koshino et al. was used to compare the grade of medial compartment cartilage lesion [35], which revealed that the frequency of cartilage regeneration was not statistically different between the two groups (Table 4).

**Table 4:** Assessment of the cartilage lesion grade at second-look operation compared with the initial operation

	Group 1 (*n* = 59)	Group 2 (*n* = 33)	*P* value ^c^
Medial compartment ^a^			0.732
Stage A (No regeneration)	18 (30.5%)	8 (24.2%)	
Stage B (Partial coverage)	32 (54.2%)	18 (54.5%)	
Stage C (Total coverage)	9 (15.3%)	7 (21.2%)	
Patellofemoral compartment ^b^			0.136
Not progressed	49 (83.1%)	23 (69.7%)	
Progressed	10 (16.9%)	10 (30.3%)	

^a^ Based on macroscopic staging system of cartilage regeneration according to Koshino et al.

^b^ Based on International Cartilage Repair Society (ICRS) grading system

^c^ Statistical significance was evaluated using Pearson’s Chi-squared or Fisher’s exact test

**Table 5:** Assessment of cartilage lesion grade of patellofemoral joint according to ICRS grade system at second-look operation compared with the initial operation

	Group 1 (*n* = 59)	Group 2 (*n* = 33)	*P* value ^b^
Preoperative ^a^			0.000
CRS grade 0	0 (0.0%)	20 (60.6%)	
ICRS grade 1	0 (0.0%)	13 (39.4%)	
ICRS grade 2	12 (20.3%)	0 (0.0%)	
ICRS grade 3	47 (79.7%)	0 (0.0%)	
Postoperative (Second-Look) ^a^			0.000
ICRS grade 0	0 (0.0%)	16 (48.5%)	
ICRS grade 1	1 (1.7%)	7 (21.2%)	
ICRS grade 2	5 (8.5%)	5 (15.2%)	
ICRS grade 3	51 (86.4%)	5 (15.2%)	
ICRS grade 4	2 (3.4%)	0 (0.0%)	
*P* value ^c^	0.071	0.007	

*ICRS* International Cartilage Repair Society

^a^ The values are given as the number of patients, with the percentage in parenthesis

^b^ Statistical significance was evaluated using Pearson’s Chi-squared or Fisher’s exact test

^c^ Statistical significance was evaluated compared with the preoperative measurement values using Wilcoxon signed rank test

## Discussion

The principal finding of the current study was that MOWHTO contributes to the progression of osteoarthritis of the patellofemoral joint regardless of the preexisting cartilage status, but this was not considered to be directly associated with clinical outcome. In addition, the clinical outcome of MOWHTO in patients with preexisting cartilage lesions of the patellofemoral joint was not inferior to those with normal cartilage. This study could suggest that the preexisting focal cartilage lesions on the patellofemoral joint, less than ICRS grade 4, would not be a hindrance to perform MOWHTO.

MOWHTO has been known to negatively affect the patellofemoral joint as a result in the change of patellar position. Decreased patellar height and an altered patellofemoral alignment increases patellofemoral contact pressure [7–9], subsequently increasing the risk of osteoarthritis progression [36]. There are several preceding studies that performed an arthroscopic assessment of the progression of patellofemoral osteoarthritis resulting from MOWHTO [11–14, 37]. However, it is difficult to conclude that MOWHTO definitely contributes to the progression of patellofemoral osteoarthritis. Although increased contact pressure of the patellofemoral joint may theoretically lead to progression of osteoarthritis in the affected joint, there are many variables to be considered. The progression of cartilage degeneration might be attributable to the normal age-dependent joint degeneration, as noted in preceding studies [12–14]. Moreover, preexisting cartilage lesions on the patellofemoral joint, which were frequently encountered during surgery, should be taken into account. Focal articular cartilage defects have been known to be a predisposing factor of osteoarthritis [16]. To determine whether MOWHTO affects the progression of patellofemoral osteoarthritis, the effect of articular cartilage status of the patellofemoral joint at the time of initial operation on the surgical outcomes should be clarified first.

Thus, the authors compared the surgical outcome of the two groups according to the cartilage status of the patellofemoral joint observed in the arthroscopic assessment performed during the initial operation. Although the two groups were classified according to the preexisting cartilage status, the proportion of the degree of patellofemoral joint osteoarthritis was not different between the two groups. This was observed not only in preoperative comparison but also at the time of second-look operation. However, osteoarthritis of the patellofemoral joint showed a tendency to progress in both groups, which was consistent with the arthroscopic assessment results. The size of cartilage lesions increased after MOWHTO in both groups, whereas the degree of change over time between the two groups was not different. The severity of cartilage lesions according to the ICRS grading system also seemed to deteriorate in both groups, but, interestingly, it was not statistically significant in the patients with preexisting cartilage lesion. It can be assumed that there was little room for arthritis progression in group 1, compared to group 2. Taking into consideration the above-mentioned findings, MOWHTO could be considered to have an adverse effect on the patellofemoral joint regardless of the preexisting cartilage status.

However, apart from the results of the radiographic and arthroscopic measurements, clinical outcomes in both groups showed overall improvement. Moreover, there was no significant difference between the two groups in clinical outcomes with respect to anterior knee pain, which demonstrates that preexisting cartilage lesions of the patellofemoral joint would not be crucial factors affecting the surgical outcomes. Various reasons may explain the discrepancy between the objective assessments and clinical outcomes in the current study. First, we had already excluded patients with symptomatic anterior knee pain and those with radiographic evidence of severe osteoarthritis on the patellofemoral joint prior to surgery. Second, the severity of preexisting cartilage lesions observed in the current study might be subtle. Although the stage of arthritis according to the Iwano classification showed a tendency to progress in both groups, severe osteoarthritis (Iwano classification stage 3 and 4) was not observed at the time of second-look operation. In addition, the source of anterior knee pain could be multifactorial including patella maltracking, malalignment of lower limb, and muscle imbalance [38]. Although osteoarthritis of the patellofemoral joint may contribute to anterior knee pain, it may not be the only factor. Therefore, the clinical outcome could not be explained by the preexisting cartilage lesion alone.

There were recent studies to investigate relevant factors that influence the progression of patellofemoral osteoarthritis after MOWHTO. Yoon et al. addressed that overcorrection, a postoperative weight-bearing line ratio > 66.3%, would lead to further progression of patellofemoral joint degeneration after MOWHTO [14]. Similarly, Tanaka et al. reported that cartilage lesions in the patellofemoral joint tended to progress after MOWHTO in patients with medial opening gap ≥13 mm or change in medial proximal tibial angle ≥9 degrees [13]. Due to the methodological differences, there are limitations in applying and interpreting the results of previous studies in the present study. However, considering the mean value of the postoperative load-bearing axis in this study, the cohorts of the present study could be considered to be at risk for further progression of the patellofemoral osteoarthritis. Nevertheless, these variables did not differ between the two groups in this study. Considering that there were no significant differences in the progression of the patellofemoral osteoarthritis between the two groups despite being at the even condition at risk of the progress of patellofemoral degeneration, preexisting cartilage lesions on the patellofemoral joint would not be a major risk factor to lead to further progression of the patellofemoral osteoarthritis. Although the present study did not examine the factors that influence the progression of patellofemoral joint degeneration after MOWHTO, it could be suggested that preexisting cartilage lesions, which were considered a risk factor performing MOWHTO, would not be a hindrance to perform MOWHTO.

### Limitations

The current study has several limitations. First, this study was based on a retrospective review, which could be associated with the risk of bias in evaluation. Second, the sample size is relatively small. However, sample size calculation indicated that the number of patients in each group was sufficient for comparison. Third, since this study was based on short-term results, it is too soon to generalize the results. Considering the increasing pattern of the cartilage lesion size of the patellofemoral joints with arthroscopic measurement in both groups, the mean follow-up period of the current study would not be sufficient to reflect the radiographic and clinical outcomes. Also, the possibility of normal age-dependent joint degeneration could not be completely excluded, since patients who did not undergo MOWHTO were not included as another control group in the current study, which was practically impossible. Fourth, data of the arthroscopic assessments were based on the medical records documented immediately after surgery, indicating that the intraoperative measurements could be associated with the risk of bias. However, since the evaluation with arthroscopic photos and movies are limited in measuring the cartilage lesion size as well as cartilage lesion depth, it would be appropriate to base it on consistently documented records which have been conducted blinded to this study. Fifth, inaccuracy in measuring the size of cartilage lesion under arthroscopic assessment should be taken into account. Although measuring the size of cartilage lesions was performed as precisely as possible, the accuracy of the measurement would still be limited since the articular surface did not have a flat contour and the border of the cartilage lesion was not clear in most of the cases. In addition, cartilage lesions of the patella and trochlea were not described separately. However, since the patellofemoral joint is a highly complicated structure consisting of a patella and femoral trochlea interacting with each other [39], the cartilage lesions of each compartment should not be evaluated individually. Therefore, the authors determined to present the result of the current study comprehensively combining both patella and trochlea lesions rather than describing them separately.

## Conclusions

MOWHTO would contribute to the progression of osteoarthritis of the patellofemoral joint regardless of the preexisting cartilage status, without an association with clinical outcomes in short-term follow-up. Consequently, based on the comparative analysis results of the subjective and objective measures, it can be suggested that preexisting cartilage lesions on the patellofemoral joint are not crucial factors affecting the surgical outcomes.

## Supplementary information

**Additional file 1.** Subgroup analysis of preoperative variables within group 1.

## Data Availability

The datasets used and/or analyzed in this study available from the corresponding author on reasonable request.

## Abbreviations

CI: Confidence interval
ICRS: International Cartilage Repair Society
IKDC: International Knee Documentation Committee
MOWHTO: Medial open wedge high tibial osteotomy
VAS: Visual analog scale
