# Supplementary material for: The effect of medial open wedge high tibial osteotomy on the patellofemoral joint: comparative analysis according to the preexisting cartilage status
Source: BMC Musculoskelet Disord. 2019 Dec 14;20:607. doi: 10.1186/s12891-019-2989-y (PMC6911704; doi:10.1186/s12891-019-2989-y)
Supplement: Supplementary file 1 — Additional file 1. Subgroup analysis of preoperative variables within group 1. [file 12891_2019_2989_MOESM1_ESM.pdf]

Additional file 1 Subgroup analysis of preoperative variables within group 1

Variable	ICRS grade 2 (n=12)	ICRS grade 3 (n=47)	*P* value
Age [a] (year)	54.8 ± 4.0	54.1 ± 5.5	0.770
Gender [b]			0.482
Male	2 (16.7%)	14 (29.8%)	
Female	10 (83.3%)	33 (70.2%)	
BMI [a] (kg/m2)	23.5 ± 7.6	26.9 ± 5.2	0.055
Load-bearing axis deviation [a] (%)	18.0 ± 9.0	19.3 ± 12.4	0.713
Preoperative Hip-Knee-Ankle angle [a] (varus,˚)	7.7 ± 2.6	7.9 ± 3.0	0.955
Medial proximal tibial angle [a] (˚)	83.6 ± 2.4	84.1 ± 2.2	0.799
Joint line convergence angle [a] (˚)	2.4 ± 1.2	3.4 ± 1.8	0.078
Posterior tibial slope (˚)	6.5 ± 4.0	5.5 ± 3.0	0.457
Preoperative Blackburne-Peel ratio [a]	0.9 ± 0.2	0.9 ± 0.2	0.118
Preoperative Caton-Deschamps ratio [a]	1.1 ± 0.2	1.0 ± 0.2	0.292
Preoperative Lateral patellofemoral angle [a] (˚)	12.1 ± 3.9	14.4 ± 4.6	0.072
Trochlear dysplasia grade according to the Dejour classification[b]			0.499
No	12 (100.0%)	44 (93.6%)	
Type A	0 (0.0%)	3 (6.4%)	
Preoperative Kellgren–Lawrence grade[b]			0.136
Grade 2	5 (41.7%)	7 (14.9%)	
Grade 3	6 (50.0%)	31 (66.0%)	
Grade 4	1 (8.3%)	9 (19.1%)	
Preoperative patellofemoral osteoarthritis stage according to Iwano's classification[b]			0.426
Stage 0	3 (25.0%)	7 (14.9%)	
Stage 1	6 (50.0%)	21 (44.7%)	
Stage 2	3 (25.0%)	19 (40.4%)	
Correction angle [a] (˚)	11.1 ± 2.3	11.0 ± 2.1	0.895
Time to hardware removal (months) [a]	20.3 ± 6.6	22.1 ± 6.2	0.406
Composition of cartilage lesion on			0.185

patellofemoral joint			
Unipolar lesion	7 (58.3%)	16 (34.0%)	
Bipolar lesion	5 (41.7%)	31 (66.0%)	
Preoperative clinical score [a]			
VAS score	72.3 ± 15.3	67.3 ± 18.3	0.463
IKDC subjective score	32.7 ± 12.6	34.1 ± 11.7	0.985
Kujala score	40.4 ± 14.0	37.8 ± 15.0	0.477

BMI body mass index, *VAS* Visual analogue scale, *IKDC* International Knee Documentation Committee

[a] The values are given as the mean and standard deviation, and compared between the groups by use of the Mann-Whitney U test

[b] The values are given as the number of patients with the percentage in parenthesis, and compared between the groups by use of the Pearson′s Chi-squared or Fisher′s exact
